# Retina Image Vessel Segmentation Using a Hybrid CGLI Level Set Method

**DOI:** 10.1155/2017/1263056

**Published:** 2017-08-03

**Authors:** Guannan Chen, Meizhu Chen, Jichun Li, Encai Zhang

**Affiliations:** ^1^Key Laboratory of Optoelectronic Science and Technology for Medicine of Ministry of Education, Fujian Provincial Key Laboratory of Photonics Technology, Fujian Normal University, Fuzhou 350007, China; ^2^Department of Ophthalmology, Fuzhou General Hospital of Nanjing Military Command, PLA, Fuzhou, China

## Abstract

As a nonintrusive method, the retina imaging provides us with a better way for the diagnosis of ophthalmologic diseases. Extracting the vessel profile automatically from the retina image is an important step in analyzing retina images. A novel hybrid active contour model is proposed to segment the fundus image automatically in this paper. It combines the signed pressure force function introduced by the Selective Binary and Gaussian Filtering Regularized Level Set (SBGFRLS) model with the local intensity property introduced by the Local Binary fitting (LBF) model to overcome the difficulty of the low contrast in segmentation process. It is more robust to the initial condition than the traditional methods and is easily implemented compared to the supervised vessel extraction methods. Proposed segmentation method was evaluated on two public datasets, DRIVE (Digital Retinal Images for Vessel Extraction) and STARE (Structured Analysis of the Retina) (the average accuracy of 0.9390 with 0.7358 sensitivity and 0.9680 specificity on DRIVE datasets and average accuracy of 0.9409 with 0.7449 sensitivity and 0.9690 specificity on STARE datasets). The experimental results show that our method is effective and our method is also robust to some kinds of pathology images compared with the traditional level set methods.

## 1. Introduction

**Figure 1:**
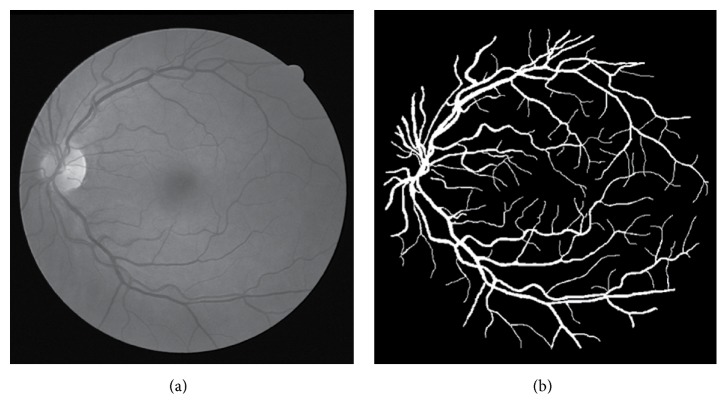
(a) Example of a retina image. (b) The related manually segmented result from the DRIVE [7] data set.

Retina image segmentation as shown in Figure 1(a), also named as fundus image, has played an important role in diagnosing the pathologies in modern ophthalmology. The morphology of the vessel is a key indicator to detect retina disease in early stages and access the severity of the disease, such as diabetic retinopathy, age-related macular degeneration, and glaucoma. Manual screening process is a much time-consuming and tedious work and the rapidly growing medical imaging technology makes us get the medical image more easily and is cheap. Thus the automatic quantification of retina vessel is extremely needed for the early diagnosis of disease in the modern CAD (computer aided diagnosis) system. The vascular tree at Figure 1(b) is a prominent element that is required for the automatic analysis of the retina image in the CAD system. However, the illumination, resolution, and field of view (FOV) in the process of acquiring the medical image and the overlapping tissue in the retina make the segmentation a challenge. In particular, due to the reflectivity of the vessels and the complexity of the tissues in retina images, the intensity inhomogeneity often occurs in the retina image. However, the segmentation of such retina image vessels is still a tough problem.

Lots of methods have been proposed in the last years. These methods are based on the different algorithm for segmentation of vessel-like structures in medical image [1–12] and can be divided into two categories, unsupervised method and supervised methods.

The first group contains methods based on unsupervised method. Sopharak et al. [4] employed optimally adjusted mathematical morphology followed by morphological reconstruction to detect exudates in nondilated retinal images. A coarse level segmentation of exudates was carried out by Jaafar et al. [7] using local variation calculation of image pixels, in order to outline the candidate boundaries. The adaptive thresholding results that have been obtained using the coarse level segmentation are further refined using a morphological operation. Welfer et al. [13] used the contrast enhanced L channel of LUV color spaces to apply morphological operations as well as H-maxima transform for exudate detection. Harangi and Hajdu [14] have proposed an active contour-based region-wise method for identifying exudates. The second group contains methods based on supervised method. Most of these methods were prone to false positives near the vascular arch. Fraz et al. [11] introduced a novel method using bagged and boosted decision trees. The decision trees are used as classification model and the results of these weak learners are combined using bootstrap aggregation. Marín et al. [10] presented a supervised method for retinal vessel detection using a Neural network (NN) scheme. A multilayer feed forward neural network is adopted which consists of an input layer, three hidden layers, and an output layer. Franklin and Rajan [15] proposed the method of retinal vessel segmentation using multilayer perceptron Artificial Neural Network (ANN). The applied neural network has three layers of one input node, five hidden nodes, and one output node. A supervised hierarchical retinal blood vessel segmentation is presented by Wang et al. [16]. Convolutional neural network (CNN) performs as a trainable hierarchical feature extractor and ensemble random forest (RF) work as a trained classifier in that work. Supervised approaches learn a model to decide whether a pixel belongs to a vessel or not with the help of manual label, where the manual segmented training images are termed as the gold standard.

Matched filter techniques [3] are the unsupervised method that uses a 2D kernel with Gaussian cross section to model the feature of the vessel in the retina image at some position and orientation. The matched filter response represents the existence of the vessel. Other unsupervised methods [17–20], which use the global and local average intensity information, employ deformable models for vessel segmentation.

In this paper, we propose a novel active contour method for the automatic segmentation of the blood vessel in the retina image. It is based on the Selective Binary and Gaussian Filtering Regularized Level Set (SBGFRLS) method [17] and the Local Binary fitting (LBF) term proposed by Li et al. [21]. In our method, the active contour is driven by the Selective Binary and local fitting term. Due to the local intensity term, the proposed model can avoid the numerical result trapped into the local minimum. The reinitialization method in our method is described in SBGFRLS model. This kind of reinitialization method simplified the computation during the evolution. Thus the proposed method is called combined global and local information method (CGLI).

Our method is tested using the following publicly available date sets: DRIVE [4] and STARE [3]. The STARE data sets are used to show the visual result of our method on pathology retina image and the DRIVE data sets are used to evaluate the performance of our methods compared with the golden standard provided.

This paper is organized as follows. We firstly review some classic models and propose our method in Sections 2 and 3. In Section 4, the segmentation result of our model on retina image is provided. The performance of our method and a comparison with the previous model and other unsupervised vessel segment methods about the segmentation result are discussed in Section 5. Finally, the summaries are given in Section 6.

## 2. Previous Methods

In the following, some basic active contour models (ACM) are reviewed. Let *Ω* ⊂ *R*^2^ represent the image space, and *I* : *Ω* → *R* represents a gray image; *C*(*p*):[0,1] → *R*^2^ represents the contour *C*. Image segmentation problem can be viewed as minimizing the energy functional and find a contour *C* in *Ω*.

### 2.1. The Geodesic Active Contour (GAC)

Based on the Fermat principle in optics, the formula of the edge-based GAC model [5] is as follows:

(1) $$\begin{array}{c} E^{gac}=\overset{1}{\underset{0}{\int }}g\left(\;\left|\;\nabla I\left(\;C\left(q\right)\;\right)\;\right|\;\right)\left|\;C^{\prime }\left(\;q\;\right)\;\right|dq, \end{array}$$

where *g* is a smooth decreasing edge stopping function (ESF)

(2) $$\begin{array}{c} g\left(\;\nabla I\;\right)=\frac{1}{\left(1+\left|\nabla K_{\sigma }*I\right|\right)}, \end{array}$$

where the ∇*K*_*σ*_*∗I* denotes the gradient of the image filtered by a Gaussian kernel *K*_*σ*_, whose standard deviation is *σ*.

Under the framework of the calculations of variation [6], the gradient flow [22] equation of (1) can be obtained and it can be used to achieve the numerical result of the minimization problem.

(3) $$\begin{array}{c} C_{t}=g\left(\;\left|\;\Delta I\;\right|\;\right)\left(\;\kappa +\alpha \;\right)\vec{N}-\left(\;\Delta g\cdot \vec{N}\;\right)\vec{N}, \end{array}$$

where *κ* is the curvature of the contour; $\vec{N}$ is the interior normal vector of the curve. *α* is a constant used to control the evolution speed.

Then the level set formulation is used as follows:

(4) $$\begin{array}{c} \frac{\partial \phi }{\partial t}=g\left|\;\Delta \phi \;\right|\left(\;div\left(\;\frac{\Delta \phi }{\left|\Delta \phi \right|}\;\right)+\alpha \;\right)+\Delta g\cdot \Delta \phi . \end{array}$$

### 2.2. The C-V Model

Chan and Vese proposed a region-based ACM model [23] based on the assumption that the image is intensity homogeneous. This method is also described as the well-known piecewise constant (PC) problem. Based on the variational level set framework [24], the CV model can be rewritten in the following form:  

(5) Ecv=∫ΩIx−c12Hϕxdx+∫ΩIx−c221−Hϕxdx+v∫ΩΔHϕxdx.

*H* in the equation is called Heaviside function and *ϕ* is the signed distance function (SDF). In practice, the *H* is often approximated by a smooth version function *H*_*ϵ*_ by

(6) $$\begin{array}{c} H_{\epsilon }\left(\;x\;\right)=\frac{\left(1+2\;arctan\left(x/\pi \right)/\pi \right)}{2}. \end{array}$$

**Figure 2:**
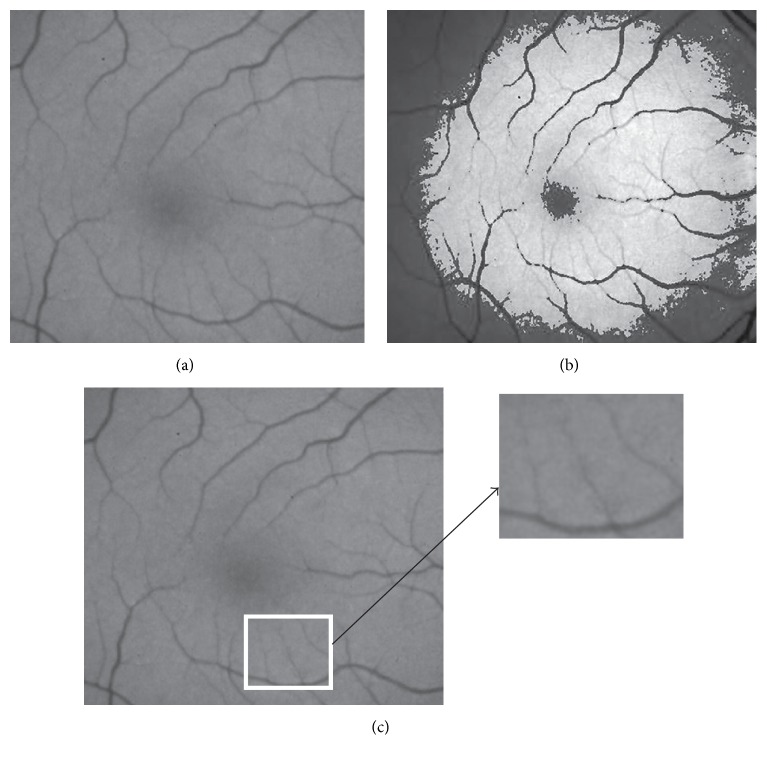
The error segmentation of the CV model in the retina vessel image and the zoomed view of the low resolution vessel. (a) The original image; (b) the segmentation result of CV model; (c) the zoomed view of vessel.

The difficulties in segmentation with retina images with CV and GAC models can be seen from Figure 2. The retina vessel in the first row in Figure 2 is the basic situation of the retina image within intensity inhomogeneity. For such images, it will not work when the thresholding method is used. In fact, no matter what threshold value is chosen, some part of the background or foreground is incorrectly identified as the foreground and the background which can be seen from the column three. At the same time, in the second row, it can be seen that the low resolution of the vessel cannot be detected in the CV model. This problem also exists in the GAC model. The example in Figure 2(c) shows the segmentation limitation of the CV and GAC models for the low resolution vessel detail in the retina image. In the meantime, the GAC model is much more sensitive to the image noise [5] which also limits its use in retina image vessel segmentation.

## 3. Proposed Methods

### 3.1. The Combination of the Global and Local Intensity Information Method

The method is based on the GAC model. Unlike the traditional GAC model, a new edge-stop function which we called signed force is used to drive the contour to the ideal boundary which takes the global and the local intensity information into consideration. In the following, the signed force based GAC will be described. The description of the edge-stop function is as follows:

(7) $$\begin{array}{c} g^{LG}=g^{L}\left(\;I\left(\;x\;\right)\;\right)+\omega g^{G}\left(\;I\left(\;x\;\right)\;\right). \end{array}$$

This new ESF is used to replace *g* at (1). The new level set formulation is obtained as follows:

(8) ∂ϕ∂t=gLGIxdiv∇ϕ∇ϕ+α∇ϕ+∇gLGIx·∇ϕ.

### 3.2. The Construction of the Local Force

**Figure 3:**
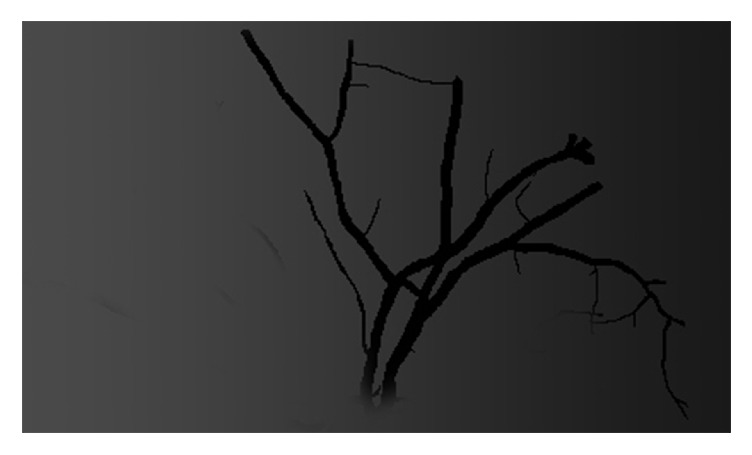
Synthetic images of intensity inhomogeneity.

In C-V model, the image is regarded as intensity homogenous. However the intensity inhomogeneity, as shown in Figures 2 and 3, is very common in retina images. So the local statistical information is required to get the segmentation result in such kind of images. In the local binary fitting (LBF) model [19], two local fitting terms *f*_1_(*x*) and *f*_2_(*x*) are used to present the intensity difference between inside and outside of the contour. The two local fitting terms make the level set method able to work even in some intensity inhomogeneity. The energy function is described as follows:

(9) ELBFϕ,f1,f2=λ1∬Kσy−xIy−f1x2·Hϕydydx+λ2∬Kσy−x·Iy−f2x21−Hϕydydx.

Due to the prosperity of Gaussian kernel function *K*_*σ*_, the contribution of the intensity *I*(*y*) to the energy at (9) decreases to zeros when *y* moves away from the center *x*. By minimizing (9), we can obtain *f*_1_ and*f*_2_ as follows:

(10) f1x=∫Kσy−xIxHϕydy∫Kσy−xHϕydy,x∈Ω,f2x=∫Kσy−xIx1−Hϕdy∫Kσy−x1−Hϕydy,x∈Ω.

This local fitting term produced by Li overcomes the limitation of the region-based level set method, but it is much more sensitive to the position of the initial contour and it is also easily trapped into the local minimum during the curve evolution. So based on the analysis above, the local fitting term is regarded as the local information term in our CGLI method; therefore our method can work effectively in the intensity inhomogeneous images and can give response to the low resolution vessel detail shown in Figure 2. The local force part of our method is based on Li's method as shown in the following:

(11) gLIx=∫Kσy−xIx−f1y+f2y/2dymax∫Kσy−xf1y+f2y/2dy.

The local fitter terms *f*_1_(*x*) and *f*_2_(*x*) represent the outside and inside intensity of the pixel point *x* near the boundary. This term makes our method able to work well in very low contrast image and is proper to segment the capillary vessel in retina image, but it is not enough. A global term is required to prevent the model from trapping into the local minimum value.

### 3.3. The Design of the Global Force

As shown in Figures 2(a) and 2(b), in inhomogeneous retina image, the CV model can only divide the image into two homogenous parts. Actually both of the parts contain the useful vessel profile. However this property can be used to avoid our model being trapped into the local minimum value only relying on the local force. So based on the global average intensity values *c*_1_ and *c*_2_ in (5), the global force is obtained as follows:

(12) $$\begin{array}{c} g^{G}=\frac{I\left(x\right)-\left(c_{1}+c_{2}\right)/2}{max\left(\left|I\left(x\right)-\left(c_{1}+c_{2}\right)/2\right|\right)},\;x\in \Omega . \end{array}$$

*c*_1_, *c*_2_ are described in (5) as the intensity inside and outside the contour.

This global force was proposed in the SBGFRLS model [17]. Equation (12) will be explained in the following. It is assumed that the intensity outside and inside the vessel is homogeneous. It is obvious that min(*I*(*x*)) ≤ *c*_1_, *c*_2_ ≤ max(*I*(*x*)). Hence, there is

(13) $$\begin{array}{c} min\left(\;I\left(\;x\;\right)\;\right)\leq \frac{\left(c_{1}+c_{2}\right)}{2}\leq max\left(\;I\left(\;x\;\right)\;\right). \end{array}$$

**Figure 4:**
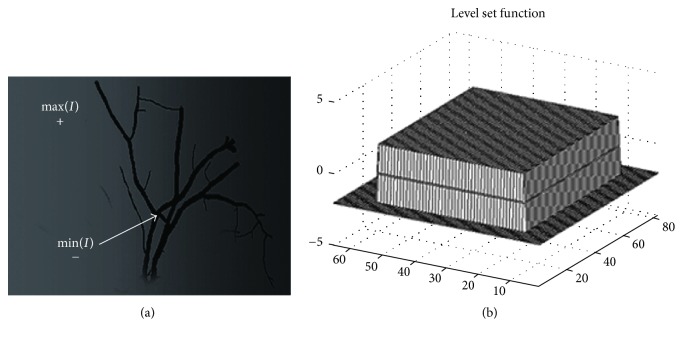
The synthetic image used for illustrating how the global force works. (a) The synthetic image; (b) the chosen level set function.

The intensity of the black vessel-like part of the image is the min(*I*(*x*)) and the gray part is the max(*I*(*x*)). Based on (13), the signed force at the black part will be minus if the contour is not at the desirable boundaries of this vessel-like part. The average intensity of the vessel-like part will be larger than min(*I*(*x*)) if the gray part could increase the average intensity inside the contour. As a result, the vessel-like part of the level set function shown in Figure 4(b) will be pulled down because of the signed force of image and the other area of the image will be pulled up. The zero level set of the *ϕ* will be the final segmentation contour.

All the energy functionals in the level set methods have an energy term to keep the result of the segmentation result contour smooth. Li et al. [19] add a regularization term in the energy to regularize the level set function; Kass et al. [25] used a curvature term to keep the contour smooth. In the GCV model a Gaussian filter is used to regularize the level set function after each iteration. The second term in the energy function at (8) is also the regularization term. The regularized way for Gaussian filter is chosen in our method, so the second term and the curvature term can be all omitted. The final formulation of our method is as follows:

(14) $$\begin{array}{c} \frac{\partial \phi }{\partial t}=g^{LG}\cdot \alpha \left|\;\nabla \phi \;\right|. \end{array}$$

As shown above, the global force can work in the intensity homogenous images. The local force based on the local fitting term only indicates the changes at the local region described by Li et al. [19]. Both of the SBGFRLS model and the LBF model have the ability to segment different kinds of images. The CGLI method takes the global force and the local force into consideration. Thus our method has the ability to deal with intensity inhomogeneous retina images and also can get subpixel accuracy and is robust to the position of the initial contour if the weight of the force is set appropriately.

### 3.4. Numerical Implementation

Here it is assumed that the target image is made of two parts, background area *Ω*_1_ and foreground area *Ω*_2_. In the traditional GAC model, it is required for initial level set function to be an SDF in order to ensure the stability during the evolution, and reinitialization is also required in the evolution. To avoid this problem, Zhang et al. [17] proposed a novel method using a Gaussian filter to smooth the level set function to regularize the level set function based on the mathematic theory of scale-space [6].

Firstly, we choose the level set function and the regularized Heaviside function *H*(*ϕ*) and its derivative *δ*(*ϕ*) in our method:

(15) ϕx,t=−ρx∈Ω0−∂Ω00x∈Ω0ρx∈Ω−Ω0,Hϵ=1+2arctan⁡z/ϵ/π2,δϵ=π−1ϵϵ2+z2.

The pseudocode of our CGLI level set method is as follows:

 
*Begin*:
 
*Img* ← *ReadImage*(*path*)
 
*ρ* ← 1
 
*K*_*σ*_ ← *Gaussian*(*size*, *sigma*)
 
*Initialize a closed contour C in the two dimension image space Ω iterationTimes←150*
 
*For times < iterationTimes*
   
$If\;\vec{x}\in \Omega \;is\;inside\;the\;contour\;C$
     
$u(\vec{x})\leftarrow \rho$
   
*Else*
   
$u(\vec{x})\leftarrow -\rho$
   
*u* ← *convolution*(*u*, *K*_*σ*_)
   
(*u*_*x*_, *u*_*y*_) ← *Gradient*(*u*)
   
(*f*_1_, *f*_2_)←* ComputeLocalBinaryValue*(*Imag*, *u*, *K*_*σ*_, *ϵ*) (10)
   
(*c*_1_, *c*_2_)←* ComputeTheRegionAverageIntensity*(*Img*, *u*, *ϵ*) (5)
   
*g*^*L*^←* ComputeTheLocalForce*(*Img*, (*f*_1_, *f*_2_), *K*_*σ*_) (11)
   
*g*^*G*^←* ComputeTheGloableForce*(*Img*, (*c*_1_, *c*_2_), *K*_*σ*_) (12)
   
*Choose*  *the*  *appropriate*  *value*  *of*  *ω*, *α*, Δ_*t*_
   
*Spf*^*LG*^ ← *g*^*L*^ + *ωg*^*G*^
   
$u\leftarrow u+\Delta_{t}(Spf^{LG}\alpha \sqrt{\left(u_{x}^{2}+u_{y}^{2}\right)})$
   
*times* ← *times* + 1
 
*End*For
 
*End*

## 4. Proposed Methods Experiment Results

### 4.1. Data Sets

The test of proposed method is using one of the popular public sets of retina fundus images called DRIVE [7]. These public data sets are popular because they provide the ground truth images that are manually segmented by different experts that make it easy for us to measure the performance of different methods.

The DRIVE database is made of 40 images, including a training set and a test set, each of which contains 20 images. The mask of the retina image of the FOV area was also provided. In the experiment, the images in the training set are used to make comparison with other methods. The performance of our proposed method is measured by comparing the automated segmentation result of the retina image with corresponding ground truth images segmented manually by experts in the training data set.

### 4.2. Performance Measurements

**Figure 5:**
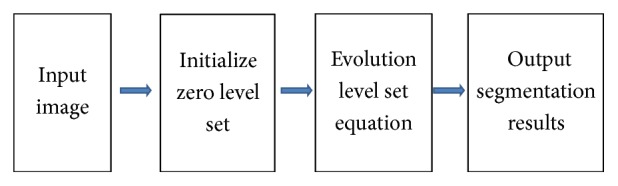
Flowchart of the proposed method.

The flowchart of the proposed method is shown in Figure 5. For each test image the final zero level set is used to obtain the vessel profile. The contour obtained using our method can divide the image pixels into two classes: vessels and nonvessels. Then, we compare the segmented binary image with their corresponding ground truth image by computing the following four performance measurements. The pixels that belong to a vessel in the ground truth image are classified as vessels and counted as true positives (TP); otherwise they are counted as false negative (FN). The pixels that belong to the background in the ground truth image are classified as nonvessels and counted as true negative (TN); otherwise they are counted as false positive (FP).

In order to compare with other vessel extraction algorithms, the accuracy (ACC), sensitivity (Se), and specificity (Sp) are calculated. These metrics are defined as follows:

(16) Acc=TP+TNN,Se=TPTP+FN,Sp=TNTN+FP,

where N = TN + TP + FN + FP.

As shown in Figure 1, the dark background outside the FOV area of the retina can be easily detected. In our experiment result, only the pixels of the FOV area are taken into consideration when we compute the performance value.

Several experiments are carried out using different sets of parameters for the application of the CGLI model. The proposed method has been tested with synthetic and real retina images. In addition, the proposed method is implemented in MATLAB 2014b on a 2.4 GHz PC. The parameters are described in Table 1.

**Figure 6:**
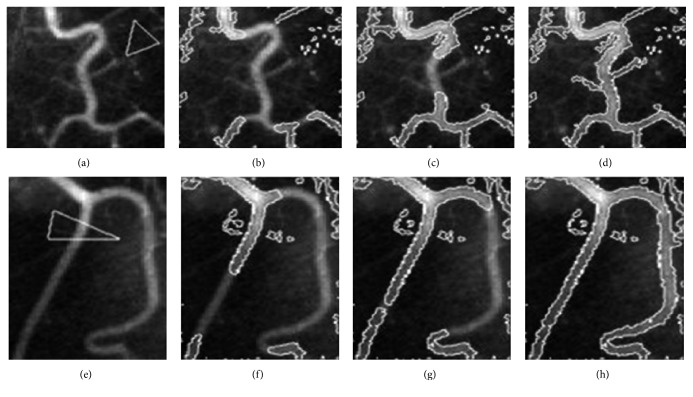
Results of our method on the X-ray images of vessels. The curve evolution process from the initial contour to the result is shown in each row. With (a)–(d) the first image of the initial contour, the 2 iterations, the 10 iterations, and the segmentation result. (e)–(h) The segmentation result of the second image.

The results for the X-ray images are shown in Figure 6, one X-ray image of vessels. The images are typical images with the intensity inhomogeneity. In these two images, it can be seen that parts of the vessel boundaries are quite weak. The segmentation result shows that our method has the ability to deal with some kinds of images with low resolution and get vessel-like structures in the images containing different tissue. Unlike the experiment result described in Li et al.'s method [19], our method does not rely much on position of the initial contour and can get more low quite weak boundaries.

### 4.3. Compared with SBGFRLS and LBF Method

**Figure 7:**
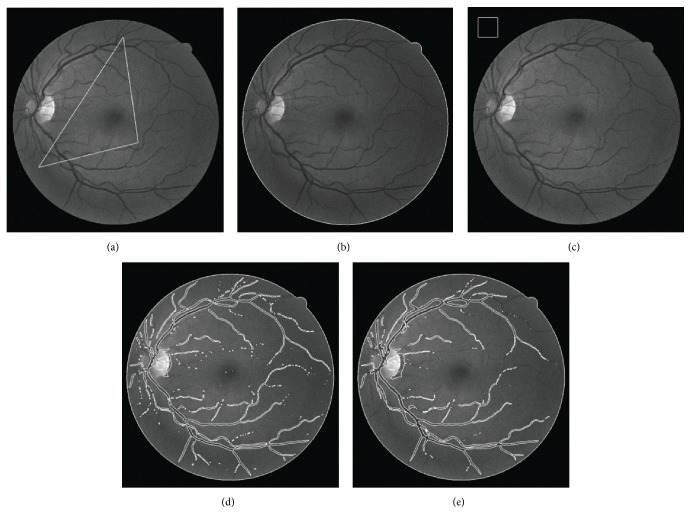
The segmentation results of the GCV and the LBF model. (a) (b) The initial contour and the result of the GCV model; the contour is marked as white. (c)–(e) The initial contour and the result of the LBF model.

**Figure 8:**
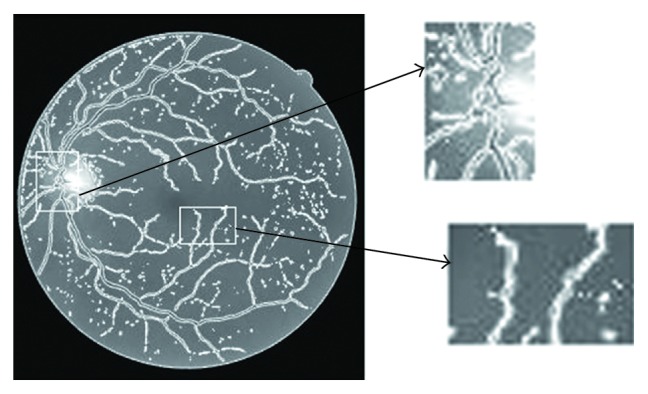
Result of our method on the real retina images and the zoomed vessel detail in the specialized optics and the low vessel rectangle.

Figure 6 shows the performance of the LBF model and SBGFRLS model separately in real retina image. It can be seen that both the SBGFRLS model and LBF model could not get the desirable vessel details by their own. In the SBGFRLS model, the curve is driven by the global intensity information, which is the same as the CV model. The segmentation result is shown at the first row. This model used a new way to reinitialize the level set function, which can reduce the time during the evolution. In the LBF model, Li used the local statistical information to overcome the intensity inhomogeneous problem. As shown in Figure 7, the curve does not converge at the describable location. The final segmentation result depends much on the position of the initial contour.  Figure 8 shows the result of the CGLI method for the real retina images in DRIVE database. It also shows the details of the segmentation. The difficulties described in Figure 6 can be solved. Not only the desirable segmentation result can be gotten, but also our method can converge at acceptable iteration times. In LBF models [18], it is not robust enough to the position of the initial position and does not converge quickly.

### 4.4. Pathology Retina Image Segmentation Details

**Figure 9:**
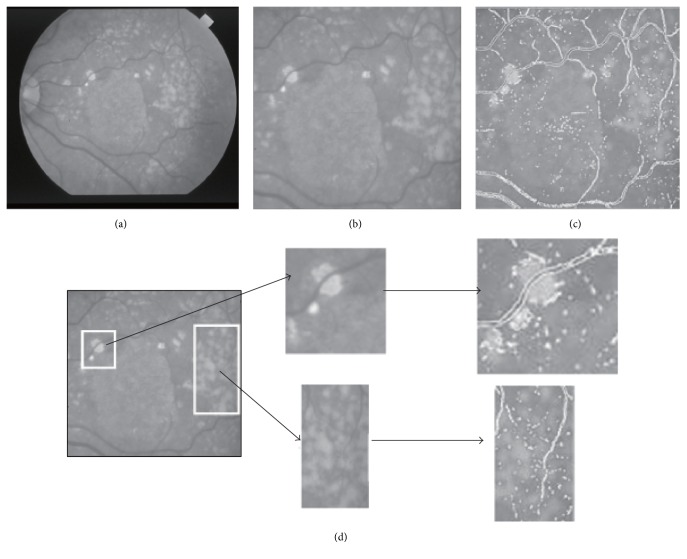
The pathology images and part of its segmentation result and some details. (a) The original pathology image; (b) part of this pathology image; (c) the segmentation result of our method; (d) the zoomed view of our segmentation result.

Our method also has the ability to deal with some pathology images, as shown in Figure 9.  Figure 9(a) is the original image from DRIVE and one part of its segmentation result from left to right. We only set one parameter *ω* = 0.2.  Figure 9(c) shows the detail of the segmentation result. It shows that our method can get the subpixel vessel profile even if the contrast of the vessel is bad as shown in the right part of the row and it also can ignore the disease area shown in the right part of the row.

## 5. Discussion

The ACM models have widely been used in image segmentation in the decays. The active contour models have lots of advantages in image segmentation when compared with traditional methods, such as match filtering and being multiscale. Firstly, active contour models can get subpixel accuracy at the object boundary. Secondly, it can be easily formulated under the framework of the energy minimization and also allows us to add the prior knowledge. Thus it can be extended in a more natural way unlike the multiscale method or match filtering method which relies on the specified models.

**Figure 10:**
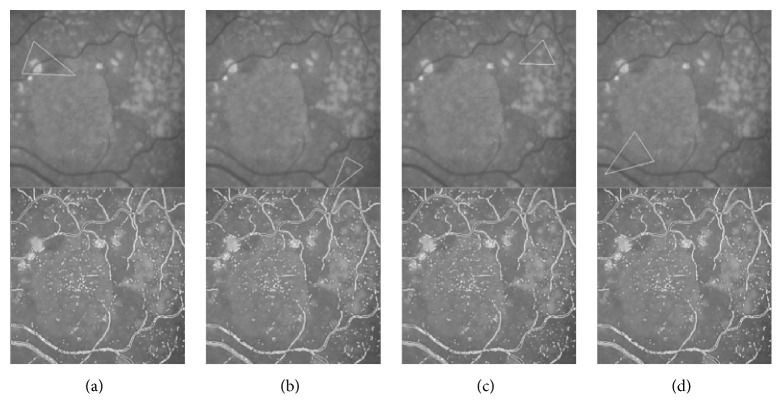
Different position of initial contour and their segmentation result.

The first row in Figure 10 shows the different position of the initial contour (white triangle) and the second row is its segmentation result. It can be seen that our method is robust to initial condition. It almost gets the same result. So our method is more robust to the position of the initial contour.

**Figure 11:**
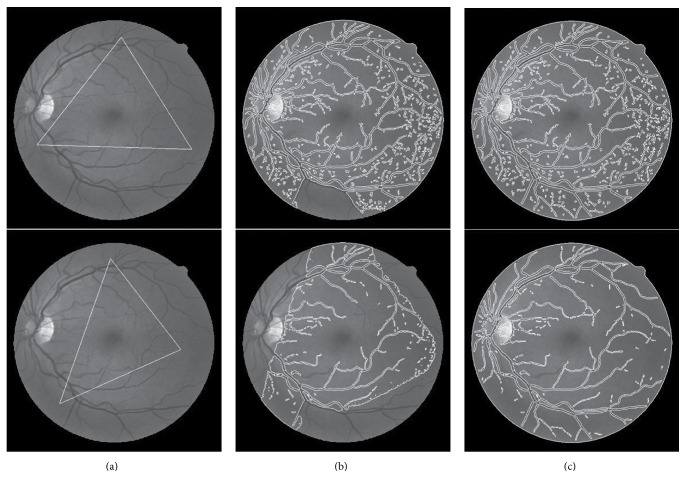
The effectiveness of parameter *ω* in the segmentation result. The first row *ω* = 0.2; the second row *ω* = 0.5. (a) The initial white color contour; (b) the contour after 20 iterations; (c) the contour convergence.

It is also necessary to talk about the parameters in our method listed in Table 1; all the parameters are predefined in our method except *ω*. *ω* decides the weight of the global force in the *g*^*LG*^ in (7). So we should set *ω* relatively small for more details and otherwise large for less details. And *ω* is the only parameter we care about in our method. Two different values of *ω* = 0.2  or 0.5 are used to show the influence of *ω*. In Figure 11, the first row *ω* = 0.2, and the second row *ω* = 0.5. Less vessel detail means larger *ω*. Generally speaking, if we want to deal with the low quality of retina images, *ω* should be set larger.

### 5.1. Compared with Other Unsupervised Method

The traditional level set method is not performing well in the retina image segmentation problem. The SBGFRLS model is a less time-consuming than level set method. The LBF method is very effective in MRI image segmentation. Both of them failed in the retina image segmentation, as shown in Figure 11; the method we proposed can run fast in image processing and it is also effective in intensity inhomogeneous images. As a result, it can perform well in retina vessel extraction. We achieve on the DRIVE data sets and compare them to other supervised and unsupervised method. The performance of our method is acceptable as shown in Table 2. Our method can also achieve better segmentation results for other medical images with intensity inhomogeneity.

## 6. Conclusion

In this paper, we propose a hybrid level set model (CGLI) based on the SBGFRLS model and the local fitting term in LBF model to segment the retina vessel automatically. We replace the original ESF function in SBGFRLS model with our new ESF function which includes the local SPF based on the local fitting term and the global SPF based on the CV model. The segmentation result of our method is controlled only by one parameter *ω*. The result we get is DRIVE (sn = 0.7358, sp = 0.9680, and acc = 0.9390) and STARE (sn = 0.7449, sp = 0.9690, and acc = 0.9409). The proposed method can segment the retina image vessel with desirable boundaries while the SBGFRLS and LBF model cannot work well in the real image segmentation. With the weight term in our method, the segmentation result can be well controlled and it also gives us the ability to deal with pathology images. Our method also has desirable quantification result when compared with other unsupervised retina image segmentation result.

## Figures and Tables

**Table 1:** The parameters used in the experiment.

Parameters	Description
*ρ*	Initialize the level set, *ρ* = 1, in our method
*σ*	Scale parameter in Gaussian kernel *σ* = 4 (default)
*ω*	The weight of the local force
Δ*t*	Time step Δ*t* = 2 (default)
*π*	*π* = 3.1415 is a constant
*ϵ*	It controls the smoothness of the Heaviside function *ϵ* = 0.5 in our method
*T*	Iteration times in the level set method
*α*	The balloon force, which controls the speed during the curve evolution in GAC method

**Table 2:** The performance of our CGLI method on the DRIVE data and STARE data compared with other methods. The result of our method is in italic.

	Method	DRIVE			STARE		
		sn	sp	acc	sn	sp	acc
Unsupervised method	*CGLI*	0.7358	0.9680	0.9390	0.7449	0.9690	0.9409
	Azzopardi et al. (2015) [1]	0.7655	0.9704	0.9442	0.7716	0.9701	0.9497
	Vlachos and Dermatas (2010) [8]	0.7468	0.9551	0.9285	0.7455	0.9544	0.9270
	Martí et al. (2007) [9]	0.6634	0.9682	0.9352	0.6701	0.9599	0.9371

Supervised method	Marín et al. (2011) [10]	NA	NA	0.9452	NA	NA	0.9344
	Fraz et al. (2012) [11]	0.7152	0.9759	0.9430	0.7311	0.9680	0.9442
